# Cancer-associated fibroblasts in gynecological malignancies: are they really allies of the enemy?

**DOI:** 10.3389/fonc.2023.1106757

**Published:** 2023-04-24

**Authors:** Julio César Villegas-Pineda, Adrián Ramírez-de-Arellano, Lesly Jazmín Bueno-Urquiza, Mélida del Rosario Lizarazo-Taborda, Ana Laura Pereira-Suárez

**Affiliations:** ^1^ Departamento de Microbiología y Patología, Centro Universitario de Ciencias de la Salud, Universidad de Guadalajara, Guadalajara, Mexico; ^2^ Instituto de Investigación en Ciencias Biomédicas, Centro Universitario de Ciencias de la Salud, Universidad de Guadalajara, Guadalajara, Mexico; ^3^ Departamento de Fisiología, Centro Universitario de Ciencias de la Salud, Universidad de Guadalajara, Guadalajara, Mexico

**Keywords:** cancer-associated fibroblasts (CAFs), tumor microenvironment, tumoral progression, pre-metastatic niche, gynecological cancers

## Abstract

Molecular and cellular components of the tumor microenvironment are essential for cancer progression. The cellular element comprises cancer cells and heterogeneous populations of non-cancer cells that satisfy tumor needs. Immune, vascular, and mesenchymal cells provide the necessary factors to feed the tumor mass, promote its development, and favor the spread of cancer cells from the primary site to adjacent and distant anatomical sites. Cancer-associated fibroblasts (CAFs) are mesenchymal cells that promote carcinogenesis and progression of various malignant neoplasms. CAFs act through the secretion of metalloproteinases, growth factors, cytokines, mitochondrial DNA, and non-coding RNAs, among other molecules. Over the last few years, the evidence on the leading role of CAFs in gynecological cancers has notably increased, placing them as the cornerstone of neoplastic processes. In this review, the recently reported findings regarding the promoting role that CAFs play in gynecological cancers, their potential use as therapeutic targets, and the new evidence suggesting that they could act as tumor suppressors are analyzed and discussed.

## Introduction

Gynecological cancer refers to any cancer that originates in women´s reproductive organs, from which cervical, ovarian, and uterine cancer stand out, and to a lesser degree but no less important vaginal and vulvar cancer (1). According to the latest Global Cancer Observatory (GLOBOCAN) report in 2020, gynecological cancers were the cause of 4,429,323 deaths and an additional 9,227,484 new cases were registered in the world (2), making them a problem of global concern in public health. Cancer-associated fibroblasts (CAFs), among other cellular and molecular components of the tumor microenvironment (TME), favor these neoplasms to occupy the first places in mortality and incidence rates (3, 4).

TME is comprised of multiple cell types, consisting of lymphatic vascular networks, adipocytes, immune, blood, and mesenchymal cells (5–7). The interaction of the different components of this cellular universe with cancer cells is essential for tumor initiation and development (7–10); it has been observed that various molecules secreted by CAFs, such as metalloproteinases (11, 12), growth factors (13, 14), cytokines (15, 16), mitochondrial DNA (17) and, non-coding RNAs (18–20); soluble released or transported in exosomes, have a promoting effect on cancer progression.

## Heterogeneous origin of a heterogeneous cell population

CAFs are a heterogeneous population of mesenchymal cells that, through the secretion of the molecules mentioned above, establish bidirectional direct communication pathways with cancer cells and other types of stromal cells such as endothelial cells and inflammatory cells (9, 21–25), promoting important cellular events for tumor progression, such as proliferation (20), angiogenesis (26), migration (27), invasion (28), tumor growth (29), epithelial-mesenchymal transition (EMT) (30), metastasis (15), and resistance to therapy (31). Being CAFs a heterogeneous cell population, it is congruent that various cell types are proposed as capable of originating them; there is extensive evidence that they can be generated from resident fibroblasts, epithelial, endothelial, and mesenchymal stem cells (MSCs) (21, 32, 33).

**Figure 1:**
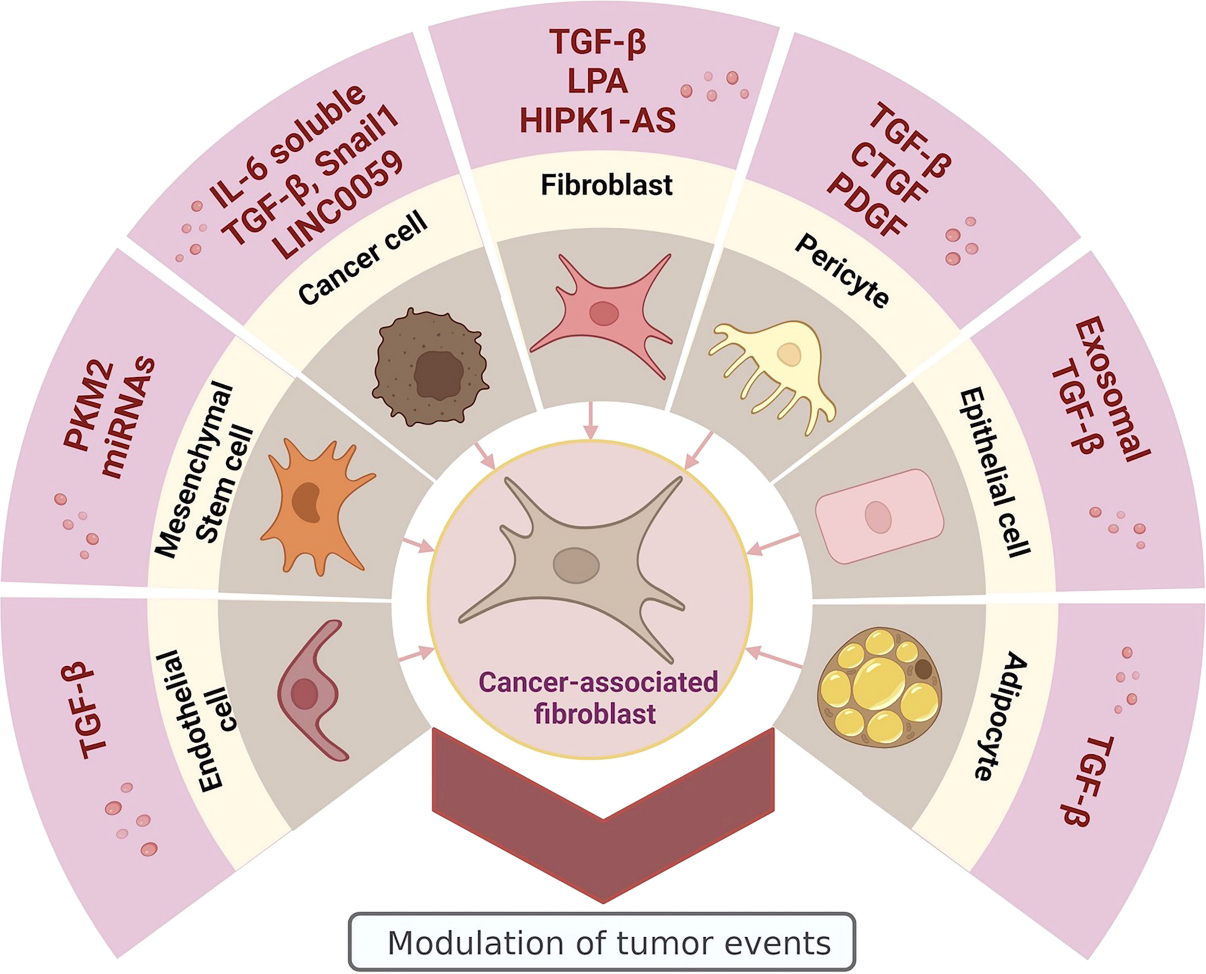
Heterogeneous origin of CAFs: promoter molecules and cellular sources. Image created in BioRender.com.

Resident fibroblasts have been proposed as the main source of CAFs (34). It has been determined that exposure to cancer cell-derived lysophosphatidic acid generates a glycolytic change and a CAF phenotype in normal and peritumoral fibroblasts (35, 36), somatic mutations in the *TP53* or *PTEN* genes would be another pathway by which physiological fibroblasts can transform into CAFs (37, 38), exosomes loaded with transforming growth factor-beta (TGF-β) secreted by cancer cells promote the generation of CAFs from fibroblasts (39). On the other hand, is the EMT, a cellular event related to carcinogenic processes that promotes the phenotypic change of healthy or cancer epithelial cells to mesenchymal cells, which has been suggested as a process of cellular dedifferentiation that generates CAFs in response to stimuli from the TME (40). These stimuli can be CAF-derived exosomes with miscellaneous content including TGF-β (30), long non-coding RNAs (lncRNAs) (41), and Snail1 transcription factor, which is characterized by facilitating tumor metastasis and promoting tumor drug resistance and recurrence (42), as well as soluble secreted interleukin 6 (IL-6) (43). Another type of transition that can also generate CAFs, is the endothelial-mesenchymal transition (EndMT), in which, TGF-β can induce the transdifferentiation of endothelial cells to fibroblast-like cells (44); this event is proposed as a unique cellular process leading to the accumulation of CAFs. Another recognized cellular origin for CAFs is MSCs (45); exosomes containing pyruvate kinase M2 (PKM2) secreted by cancer cells have been shown to promote the transition from MSCs to CAFs (46). The load of the CAF-generating exosomes can be quite diverse; in a study carried out with exosomes derived from chronic lymphocytic leukemia cells, it was determined that the miscellaneous exosomal content was enriched with antiapoptotic proteins, angiogenic factors, RNA processing proteins, oncogenes, heat shock proteins, and microRNAs (miRNAs) (47). These pro-tumor molecules generated a permanent source of CAFs by inducing an inflammatory phenotype in stromal cells and increased their proliferation and migration. In addition, they promoted angiogenesis and tumor growth in a mouse tumor model (32, 47). Other cells that have also been proposed as the origin of CAFs are adipocytes (48, 49), smooth muscle cells (44), and pericytes (50), which in response to TME molecules, such as TGF-β (48), platelet-derived growth factor (PDGF) (51), and connective tissue growth factor (CTGF) (52) acquire a CAF-like phenotype through cellular transdifferentiation, and consequently, participate in the promotion of carcinogenesis and tumor development (**Figure 1**).

Although CAFs are not themselves cancer cells and independently of their heterogeneous origin, they promote tumor growth and maintenance, suggesting that their participation in cancer progression is essential. Therefore, their study has become necessary to understand comprehensively the genesis and development of malignant neoplasms and evaluate if this cell population can be a potential therapeutic target in patients with gynecological cancers.

## Signaling pathways involved in the generation of CAFs

The generation of CAFs from different cellular origins is possible due to the activation of various signaling pathways. In the case of CAFs originating from MSCs, it has been observed that the CAF phenotype is acquired through activation of the Janus Kinase/Signal transducer and activator of transcription 3 (JAK/STAT3) signaling pathway induced by TGFβ1, promoting migration and invasion by upregulating the expression of N-cadherin and vimentin and down-regulating E-cadherin (53). Biffi et al. reported that the cytokine IL-1 secreted by tumor cells can also activate this pathway and that the specific formation of inflammatory CAFs is promoted through the participation of nuclear factor kappa B (NF-kB) (54).

The suppressor of mothers against decapentaplegic (SMAD) pathway is also involved in the generation of CAFs; in a study, it was found that human dermal fibroblasts treated with TGF-β overexpressed p-Smad3, which in turn increased the expression of α-smooth muscle actin (α-SMA) and integrin subunit α11, characteristic markers of the CAF phenotype. While fibroblast growth factor 2 (FGF2) treatment downregulated CAF activation genes, including *ACTA2* and *ITGA11* (55). These results show that the CAF phenotype is not irreversible and that the SMAD pathway induced by TGF-β is fundamental for cell differentiation to CAFs.

CAFs favor tumor progression through the secretion of exosomes containing miRNAs. It has been found that CAFs are able to secrete exosomes containing miR-92a-3p, which promote stemness, EMT, metastasis, and chemotherapy resistance, by activating the WNT/β-catenin signaling pathway in cancer cells (56). CAFs can also be targets of exosomal miRNAs. Breast cancer cells secrete exosomes loaded with miR-105, which CAFs can take up. Once inside the cell, miR-105 binds to the myelocytomatosis oncogene (MYC) negative regulator, *MXI1*, activating MYC signaling and promoting the expression of genes related to glucose (*HK2*, *LDHA*, *LDHB*) and glutamine (*GLS*) metabolism and for metabolite transportation (*SLC2A1*, *SLC16A1*, *SLC16A3*, and *SLC1A5*) converting CAFs into an energy source for cancer cells in the TME (57). It has also been observed that CAFs under hypoxic conditions, a common feature of TME in most tumors due to nutrient deficiency, increase their glycolytic activity *via* oxidized ataxia-telangiectasia mutated protein kinase (ATM) and glucose transporter 1 (GLUT1) phosphorylation. This hypoxia-induced glycolysis promotes lactate secretion, enhancing the activation of TGFβ1/p38 mitogen-activated protein kinase/matrix metalloproteinase 2/9 (MAPK/MMP2/9) signaling in breast cancer cells, promoting the invasiveness of tumor cells (58). Extracellular matrix (ECM) remodeling is one of the characteristics of CAFs, this mainly proteolytic event facilitates the invasion and metastasis of tumor cells; some of the proteins secreted by CAFs are enzymes such as MMP11, which is a metalloproteinase belonging to the endoproteases family, responsible for the degradation of the ECM (59). MMP11-positive CAFs have been found enriched in the stroma of invasive ductal carcinoma, being associated with tumor progression and poor prognosis (60). On the other hand, it was shown that miR-139 inhibits tumor growth and metastasis of gastric cancer cells by decreasing the expression of MMP11 (61).

CAFs exert an immunomodulatory role in TME (62); through the secretion of IL-6 and granulocyte-macrophage colony-stimulating factor (GM-CSF), they induce the differentiation of monocytes towards M2-like tumor-associated macrophages (TAMs), and the immunosuppressive environment generated facilitates tumorigenesis and metastasis (63). This same immunomodulatory and immunosuppressive role has been observed in CAF-like cells differentiated from MSCs co-cultured with esophageal squamous cell carcinoma cells (64). CAFs have paracrine communication with tumor cells, so their activation is the feedback of several signaling pathways; in addition, they regulate the tumor microenvironment through the secretion of soluble factors involved in immune modulation. These events reveal the triple cross-talk between cancer cells, CAFs, and monocytes to promote tumor progression.

## Classic CAF markers

CAFs are spindle-shaped cells that reside around the tumors, negative to markers for other cells, such as endothelial, epithelial, or leukocytes (65). They express different markers, making them different from normal fibroblasts, such as the case of α-SMA, fibroblast activation protein (FAP), fibroblast-specific protein 1 (FSP1), and platelet-derived growth factor receptor (PDGFR) (66–68). Besides, some other proteins are usually expressed in CAFs, like collagen 11-α1 (COL11A1), microfibrillar-associated protein 5 (MFAP5), and asporin (69). Some markers are shared between subpopulations, making it harder to isolate and study them, and their expression is variable depending on the CAF subtype (70), for example, FAP, PDGFR-α, PDGFR-β, podoplanin, integrin-β, and caveolin-1 (65).

α-SMA belongs to the actin family, which is important for cell structure and motility (69). Initially, it was thought to be the most representative marker for CAFs; however, not all CAFs express this protein. It has also been associated with prognosis; for instance, a high expression of α-SMA correlates to lower overall survival in breast cancer (71).

FAP has been proposed as one of the primary markers of CAFs being overexpressed in 90% of carcinomas, such as melanoma, colorectal, breast, ovarian, bladder, and lung (72, 73) and with high therapeutic potential (74). This membrane protein is commonly used as a CAF-activation marker because it has been found to be highly expressed in stromal cancer tissue (75–78). Moreover, some reports conclude that FAP is also expressed in cells during the EMT and carcinoma cells (79, 80). Vimentin is an essential protein involved in the cytoskeleton network. Because a mesenchymal phenotype characterizes fibroblasts, vimentin is expressed in all types of fibroblasts. The strength of this biomarker is reduced because various cells, such as pericytes and adipocytes, express vimentin and those undergoing EndMT and EMT (69, 81, 82).

Molecules such as MFAP5 and COL11A1 are novel markers suggested as promising biomarkers for CAFs (69, 83–86); even though they are suggested to be extremely specific, they are not commonly used in the literature. Newly discovered biomarkers are essential to classify CAFs in different cancer types or according to their signature or specific actions such as chemoresistance, immunomodulation, or ECM remodeling (87).

Even though most biomarkers have pro-tumor effects, a few exert anti-tumor actions; cluster of differentiation 146 (CD146) and caveolin-1 are examples of this type of biomarkers. Two CAF subpopulations can be identified by the presence/absence of CD146, which has been correlated to tamoxifen sensitivity, and low expression of caveolin-1 in CAFs is associated with poor prognosis in breast cancer (88, 89).

The heterogeneity of CAFs might reflect the different stages of their activation from a common cell, according to the signals they receive. For instance, normal fibroblasts can become CAFs after receiving signals from the TME (90). Furthermore, some CAFs might stop expressing their markers, indicating that the process is reversible (91). Observations like these suggest that CAFs are not a cell type but a transitional state of fibroblasts, making the difference between both cells a functional matter rather than a marker one. However, some authors have classified CAFs into different subpopulations depending on the expression of their markers and their tumor context. In pancreatic ductal adenocarcinoma, they subdivide them into two main populations characterized by their location and functionality: activated CAFs close to the tumor with a myofibroblast phenotype (myCAF) express high levels of α-SMA and low levels of cytokines, whereas inflammatory CAFs (iCAFs) far from the tumor express low levels of α-SMA and high levels of cytokines (92).

In addition, in a study conducted on breast cancer, CAFs were mainly classified into four subclasses CAF-S1 to CAF-S4; each of the subpopulations was characterized by the expression of markers and their accumulation in the molecular subtypes of breast cancer. Subclass CAF-S1 positive for FAP and α-SMA and subclass CAF-S4 FAP negative and α-SMA positive were detected enriched in TME triple-negative breast cancer, and of these two subpopulations, CAF-S1 stands out for having an immunosuppressive role by mainly recruiting regulatory T cells through the secretion of C-X-C motif chemokine ligand 12 (CXCL12) (93). CAF-S1 and CAF-S4 have also been associated with breast cancer metastasis; CAF-S1 promotes tumor cell migration and EMT initiation, and CAF-S4 promotes cancer cell invasion and motility (94). Some transcription factors are present in CAFs and may regulate pro- or anti-cancer properties, depending on the context and the cell type (54, 95–97). Molecules such as heat shock factor 1 (HSF1), signal transducer and activator of transcription 3 (STAT3), MYC, and yes-associated protein (YAP) might be involved in specific signatures of CAFs and reprogramming (54, 98, 99). Nowadays, new CAF-associated markers are being identified, due to new technologies such as single-cell sequencing, which opens the landscape for new targeted therapy approaches directed to CAF populations, such as immunotherapy directed to CAFs or the cytokines and chemokines derived from them (40).

## Impact of CAFs on gynecological cancers

### Ovarian cancer

Ovarian cancer (OC) is considered the most lethal gynecological neoplasm due to the high number of deaths in proportion to its incidence. The latest report from the International Agency for Research shows that in 2020 it was the cause of 313,959 new cases and 207,252 deaths in the world (2). The lack of laboratory tests and the absence of pathognomonic symptoms that evidence the early stages of OC, cause this neoplasm to be diagnosed in late stages (III and IV), when cancer has already metastasized, compromising other organs and generating a poor prognosis. The low 5-year survival rate of patients with late-stage epithelial ovarian cancer (30%) is a consequence of the rapid progression of this disease (100). OC is promoted by factors of the TME, among them CAFs stand out. CAFs are the predominant cell type in the TME of different neoplasms; in OC their presence is associated with increased migration and invasion of cancer cells (101–103), extracellular matrix remodeling (104), spheroid formation (105), tumor growth promotion (106), metastasis (107, 108), angiogenesis (109), chemoresistance (110) and poor prognosis for OC patients (111, 112), cellular events that promote neoplastic development and make them a good therapeutic candidate.

In OC, as in other neoplasms, it has been shown that TGF-β plays an important role. This growth factor is secreted by cancer cells, inducing the differentiation of normal fibroblasts into CAFs. The sphingosine kinase 1 (SphK1) enzyme is responsible for mediating TGF-β signaling through the transactivation of sphingosine 1-phosphate receptor 2 (S1PR2) and S1PR3, leading to p38 MAPK phosphorylation and the consequent acquisition of the CAF phenotype. *In vivo* assays demonstrated the participation of SphK1 in tumorigenesis, promoting tumor growth and metastasis (113). CAFs can also secrete TGF-β, which activates paracrine signaling, promoting the progression of OC due to the expression of various genes, including *MMP11* and *MMP13*, promoting the metastasis of ovarian cancer cells to adjacent or distant sites. Overexpression of *MMP13*, along with *CGA*, *EPHA3*, *PSMD9*, *PITX2*, and *PHLPP1*, has been associated with poor response to platinum-based chemotherapy in patients with high-grade serous ovarian cancer (HGSOC), the most aggressive form of ovarian cancer (114). Li et al. conducted a study with CAFs isolated from stage IIIC OC patients in which they determined that CAFs, through the secretion of exosomes loaded with TGF-β, can promote various pro-tumor events such as migration, invasion, and EMT of OC cells through the activation of SMAD signaling pathway. Additionally, in a model of xenotransplanted mice, they observed that the co-inoculation of ovarian cancer cells and CAFs favored the generation of tumors compared to animals inoculated only with ovarian cancer cells (30), these results suggest the pro-tumor role of CAFs and TGF-β in the TME of OC.

CAFs promote tumor development by providing the cells of the TME with the necessary signals to proliferate and enhance angiogenesis, through the overexpression of NF-kB, IL-6, cyclooxygenase-2 (Cox-2), and CXCL1, molecules known to have pro-tumorigenic activity (115). It has also been observed that CAFs can overexpress nicotinamide N-methyltransferase methyltransferase (NNMT), an epigenetic regulatory molecule that acts through hypomethylation of DNA, RNA, or histones *via* attenuation of the S-adenosyl methionine/S-adenosyl homocysteine (SAM/SAH) ratio. This enzyme is essential for the expression of CAF markers, the secretion of pro-tumorigenic cytokines, and oncogenic ECM. CAFs overexpressing NNMT promoted OC migration, proliferation, EMT, and metastasis (116).

CAFs not only maintain cellular communication with cancer cells, but it has also been observed that in the TME of OC, communication between CAFs and cancer-associated macrophages (CAMs) leads to cancer cell invasion and metastasis. CAFs, through secretion of IL-33, lead to the expression of M2 macrophage marker genes in human blood‐derived monocytes; in turn, CAF‐induced CAMs increase the invasion and migration of OC cells and upregulate the EMT marker genes (117). To promote tumor progression, CAFs also maintain communication with endothelial cells, upregulating the lipoma-preferred partner (LPP) gene in microvascular endothelial cells (MECs); this event increases the formation of focal adhesions and stress fibers and, consequently, endothelial cell motility, intratumoral microvessel leakiness and chemoresistance to paclitaxel. Mechanistically, MFAP5 derived from CAFs promoted the expression of LPP in microvascular endothelial cells by activating the focal adhesion kinase/extracellular signal-regulated kinase/cAMP responsive element binding protein (FAK/ERK/CREB) signaling network, generating resistance to paclitaxel by increasing cell migration and their focal adhesions, which weakens blood vessels causing paclitaxel to leak before being released to OC cells (118). MFAP5, overexpressed and secreted by CAFs into the EMC, binds to the αvβ3 integrin of OC cells. This ligand-receptor union activates the calcium-dependent FAK/ERK/CREB signaling pathway, leading to overexpression of troponin C type 1 (TNNC1), increasing cell mobility by promoting the formation and rearrangement of the F-actin cytoskeleton. TNNC1 has been proposed as a biomarker for poor prognosis for HGSOC. *In vivo* assays, MFAP5 promoted tumor progression by increasing angiogenesis, tumor growth, invasion, and metastasis (84) and MFAP5 blockade using monoclonal antibodies was able to inhibit fibrosis and enhance chemosensitivity in mouse models, generating tumor suppression (119). These results propose MFAP5 as a new and promising therapeutic target in patients with OC.

FAP, a classic marker of CAFs, has been associated with recurrence in patients with epithelial OC after treatment with neoadjuvant chemotherapy; strongly FAP-positive tumor parenchyma and stroma were seen in tumors from patients with high recurrence rates (OR: 15.95) while FAP-negative tumors were seen in patients with lower recurrence rates (OR: 0.086) (120). FAP could be used clinically as a negative prognostic marker for patients with OC. At the same time, it could be the target of inhibitory molecules that help counteract its pro-tumor action in the TME.

CAFs manifest their pro-tumorigenic behavior across a broad molecular spectrum; myristoylated alanine-rich C-kinase substrate (MARCKS) is overexpressed in CAFs and has been strongly associated with expression of the classic CAF marker α-SMA. MARCKS participates in the activation, proliferation, chemotherapeutic resistance, and migration of CAFs, its silencing in CAFs decreases the proliferation and migration of OC cells and tumor growth, together MARCKS derived from CAFs facilitates OC metastasis (121), for which it is suggested that MARCKS could be an attractive target for the therapy of patients with OC. Similarly, periostin (POSTN) is enriched in the OC stroma, particularly in CAFs from HGSC; its overexpression was correlated with reduced overall survival. POSTN increased OC cell migration and invasion by functioning as an αvβ3 integrin ligand, activating the PI3K/Akt pathway and promoting the EMT (122); stromal-derived POSTN may be a potential therapeutic target given that it participates in the remodeling of the pre-metastatic niche (PMN) of OC. CAF-derived CXCL12 also induces EMT *via* the CXCR4/Wnt/β-catenin pathway; CXCL12 expression was associated with cisplatin chemoresistance in OC patients (123). These findings, together with other evidence, propose CAFs as a modulator cell type with a decisive role in generating chemoresistance, angiogenesis, remodeling, and immunomodulating the TME and the PMN (124–126).

Cancerous development has a high energy demand, glucose being the main molecule to obtain ATP and the high consumption of glucose allows cancer cells to maintain their high rate of uncontrolled growth. CAFs favor glucose uptake by OC cells modulating the activation of key enzymes in cellular glycogen metabolism, which increases the energy available to enhance the migration and invasion of OC cells. In human samples, it was observed that the greater the progression of the OC, the lower the glycogen level in the tissue and the greater the number of CAFs. The authors demonstrated that glycogen mobilization in cancer cells depended on p38α MAPK activation in CAFs by TGFβ released by the cancer cells, leading to increased secretion of the cytokines CXCL10, IL-6, and IL-8 (127, 128).

The difficulty of diagnosing ovarian cancer in its early stages is mainly due to the anatomical site where the ovaries are located and the lack of early biomarkers; in search of new candidates for biomarkers, Lawrenson et al., have non-invasively detected natriuretic peptide B (NPPB), a hormone initially unrelated to ovarian neoplasia and that is secreted from cardiac ventricular myocytes in response to myocardial stretch and stress, in the stroma of 60% primary OC tissues and the blood of 50% of women with OC (129). Although NPPB was expressed by only 28% of early-stage OCs, it could be part of the design of a panel of early biomarkers that together increase their sensitivity and specificity.

There are several reports in which it is shown that CAFs non-coding RNAs (ncRNAs) promote the development of various cancers (130). A predictive functional analysis identified 39 lncRNAs differentially expressed in CAFs compared to fibroblasts, 17 lncRNAs were up-regulated in CAFs and 22 lncRNAs were downregulated. The *in silico* analysis allowed to associate this signature of lncRNAs with multiple pathways in OC metastasis (131), the findings of this study suggest that lncRNAs modulate the CAFs phenotype and that the upregulated or deregulated expression of these ncRNAs favors OC metastasis and progression. An example of deregulated miRNA in CAFs is miR-124, a highly conserved tumor suppressor ncRNA, and highly expressed in fibroblasts. Downregulation of miR-124 is associated with tumorigenesis, tumor progression, and poor prognosis in OC patients. Its decrease or inhibition promotes cell migration, which leads to the acquisition of an aggressive cell phenotype. The cellular effects of miR-124 are exerted by inhibiting SphK1, which catalyzes the phosphorylation of sphingosine to form sphingosine 1-phosphate (SPP), a key sphingolipid signaling molecule involved in cell growth, survival, differentiation and motility (132). Additionally, it has been observed that exosomes loaded with miR-98-5p secreted by CAFs increase cell proliferation, inhibit apoptosis, and promote cisplatin resistance in ovarian cancer cells by downregulating cyclin-dependent kinase inhibitor 1A (CDKN1A) (133).

Despite the extensive evidence on the pro-tumor role of CAFs in the TME, favoring tumor growth and metastasis (134, 135), there are some reports that suggest an anti-tumor role for these cells of heterogeneous origin. Colvin et al. demonstrated that CAFs are capable of secreting ncRNAs with anti-tumor properties, such as *MIR155HG*. This lncRNA could be considered as a biomarker of good prognosis for patients with OC, since its overexpression has been associated with more remarkable patient survival, T cell activation, antigen processing and presentation, and with the enriched infiltrate of immune cell anti-tumor within the tumor, highlighting plasma cells, various subpopulations of T cells, M1 macrophages, and eosinophils (136). This finding opens the possibility to the existence of subpopulations of CAFs with a clear anti-tumor role; modulating, targeting, or enriching these cell subpopulations in the TME could help fight early-stage cancer development.

### Endometrial cancer

Fibroblasts are present in the endometrial stroma, which surrounds the endometrial glandular epithelium. These fibroblasts can be transformed into CAFs by endometrial cancer cells, and once this transformation occurs, CAFs gain some characteristics that support the development of cancer (137). The accumulating evidence shows that CAFs can communicate with microenvironment cells through extracellular vesicles (EV)/exosomes, which transport several molecules, including miRNAs (138). Some miRNAs have a regulatory activity, either pro- or anti-tumor; being the latter the case of miR-320a. In endometrial cancer (EC), miR-320a inhibits the proliferation of EC cells, downregulates the expression of hypoxia-inducible factor 1 alpha (HIF-1α) in EC cells, and inhibits HIF1α/VEGFA axis (139). This axis, in particular, is fundamental in EC because its inhibition is suggested to have an improved radiosensitivity in EC cells (140). Even though CAFs can directly transfer miR-320a into EC cells, they normally express this tumoral suppressor miRNA to a lower extent (139), encouraging debate on the pro- or anti-tumor role of CAFs. miR-148b has also been reported to have anti-tumor effects. Li et al. concluded that loss of exosomal miR-148b promotes metastasis *in vitro* and *in vivo*, and it induces EMT and invasion of endometrial cancer cells *via* the relief of the suppression of DNA methyltransferase 1 (DNMT1) (141). However, other miRNAs have been reported to be associated with important features of cancer, like miR-22, which has been associated with resistance to tamoxifen treatment in patients with breast cancer (142). Taken altogether, miRNAs in EV derived from CAFs could be evidencing the existence of diverse populations of CAFs, both anti- and pro-tumor depending on the type and amount of miRNAs they contain, that inhibit or promote events which favor malignancy development, making them a focus for targeted treatment in EC.

A new approach for therapeutics is directed to the interaction between lncRNAs and miRNAs, also called Competing Endogenous RNA (ceRNA) (143). Several reports suggest that this regulation may be vital for CAFs to support growth and metastasis in endometrial cancer. Long non-coding RNAs have also been involved in EC development, such as the case of nuclear enriched abundant transcript 1 (NEAT1), which was transferred to the endometrial cell lines HEC-1A and RL95-2 through the exosomes derived from CAFs and promoted tumorigenicity *in vivo*, increasing the levels of YKL-40, an EC marker. The opposite effect was observed by miR-26a/b-5p, which decreased the YKL-40 and STAT3 expression. NEAT1-overexpressing CAFs downregulated the expression of miR-26a/b-5p, and the opposite effect was observed by CAFs that did not express NEAT1 (25); this might represent an example of pro-tumor ceRNA, it is necessary to design and carry out clinical studies where its anti-tumor uses are evidenced by inhibiting miRNAs that promote cancer progression. Studies suggest that PTTG is a key modulating factor in carcinogenesis because of its involvement in processes like cell cycle progression, angiogenesis, malignant transformation, and metastasis (144, 145). Wang et al. reported that CAFs could induce the expression of PTTG in EC and therefore contribute to the carcinogenesis progress. The pituitary tumor transforming gene (PTTG) expression was evaluated by RT-PCR and Western blot showing a significant increase in the mRNA and protein levels in the EC cells cocultured with CAFs compared to those with normal fibroblasts. They also observed that CAFs increased EC cell invasion and migration and induced EMT in EC cells by regulating PTTG in an *in vitro* model (146). CAFs derived from human endometrium induced a higher proliferation of endometrial cells than normal endometrial fibroblasts. AKT and ERK were highly phosphorylated in ECC-1 cells after being treated with medium conditioned of CAFs, which might indicate that PI3K and MAPK are responsible for the observed proliferation effect (147).

Similar to other cells, CAFs can respond to hormones. Estradiol (E2) and progesterone are essential hormones in regulating the menstrual cycle and mitogenic responses. Unopposed E2 can lead to endometrial hyperplasia due to its proliferative effects, while progestins are protective, leading to cell differentiation of endometrial glands, in contrast to E2; in fact, synthetic progestins are indicated in the treatment of complex atypical hyperplasia and low-grade type I EC, suppressing the actions of E2 (148–151). TME of EC has been documented to express low levels of progesterone receptor and estrogen receptor alpha (ER-α) (152), which may cause the lack of responsiveness of the CAFs to these hormones. Besides, this may lead to altering the paracrine regulation in EC. Progesterone induces the release of PEDF in CAFs and endometrial stromal fibroblasts, the expression of this potential growth inhibitor is associated with several human cancers; whereas high concentrations relate to a good prognosis, low ones are known to be pro-tumorigenic (153).

Another reported effect of CAFs is the suppression of natural killer (NK) cells´ cytotoxic activity. Inoue et al. reported a decrease in the killing activity of NK cells, which was cell-cell contact-dependent and associated with a diminished cell-surface Poliovirus receptor (PVR) expression, an essential ligand for DNAX accessory molecule-1 (DNAM-1), an activating receptor present on NK cells (137). Finally, CAFs may support EC development by the secretion of proinflammatory cytokines such as IL-8, IL-6, monocyte chemoattractant protein-1, and VEGF, and the tumoral growth stimulation through the stromal cell-derived factor–1a/CXC chemokine receptor 4 axis (147, 154, 155). More research must be conducted to identify the CAF secretome in the EC context.

### Cervical cancer

CAFs have fundamental participation in the progression of cervical cancer (CC); cancer cells and CAFs have paracrine communication through various soluble factors such as proteins, IncRNAs, and miRNAs, among other molecules. Wnt2B is a protein secreted by cancer cells in exosomes, which participates in the differentiation of fibroblasts towards CAFs, activating the Wnt/β-catenin signaling pathway, inducing transcription through LEF/TCF and, consequently, promoting tumor growth in nude mice (156). In CC, one of the generating factors of CAFs is IncRNAs; homeodomain-interacting protein kinase 1 antisense RNA (HIPK1-AS) overexpressed in HeLa conditioned medium induced the differentiation of normal human cervical fibroblasts towards CAFs, increasing the expression of FAP, IL-6 and α-SMA; HIPK1-AS modulation is reversible because its deletion down-regulates CAF activation; the mechanisms of HIPK1-AS are not yet fully elucidated (157). In addition to being a CAFs-generating molecule, TGF-β is secreted by itself, promoting migration and invasion of CC cells, essential events for tumor growth and metastasis (28, 158, 159). These findings show the important communication between cancer cells, fibroblast, and CAFs to facilitate metastasis in CC.

In the TME of the CC, the two-way communication between CC cells and CAFs is constant and favors the development of the disease. PDGF-BB secreted by cancer cells induces the expression of heparin-binding epidermal growth factor-like growth factor (HB-EGF) by CAFs, through activation of PDGFRβ, which in turn activates epidermal growth factor receptor (EGFR) in CC cells promoting cell proliferation and tumor growth, in *in vitro* and *in vivo* models, respectively. In patient´s tissues, it was observed that HB-EGF expression is increased during cervical carcinogenesis (160), suggesting that it participates in carcinogenesis, maintenance, and progression of the CC.

microRNAs contained in extracellular vesicles derived from CAFs facilitate tumor growth in *in vivo* models, such as the case of microRNA-10a-5, which is capable of inducing angiogenesis *via* activating the Hh pathway by inhibiting TBX5 and promoting the expression of VEGF (18). In athymic nude mice, it was observed that the co-inoculation of CAFs and CC cells, in addition to generating tumors, caused metastasis in 40% of lymph nodes, while the inoculation of only cancer cells did not generate metastasis (161). EMC proteins also contribute to the progression of CC generated by CAFs. *In vitro* assays showed that CC cells mainly express integrin α6β4 laminin receptors and, are conveniently capable of inducing laminin expression by CAFs, event that, together with the secretion of MMP-7 by CC cells for degradation of the basement membrane, favors the invasion of CC (162). These results show the cellular intercommunication in the TME and the essential role played by CAFs in the pro-tumor remodeling of the EMC.

Various subtypes of CAFs associated with the progression of this neoplasm have been reported; periostin-positive CAFs are involved in lymph node metastasis and with poor survival of CC patients due to the promotion of the permeability of lymphatic endothelial barriers through activation of integrin-FAK/Src-VE-cadherin signaling pathway in lymphatic endothelial cells, which decreases the expression of VE‐cadherin favoring metastatic dissemination (163). Because CAFs are the most abundant stromal cells in TME, they are a good therapeutic target; nanoparticles are a promising tool since they can be targeted with great cellular specificity, *in vitro* studies showed that HeLa cells and CAFs have higher intake and retention of gold nanoparticles with surface functionalized with both polyethylene glycol (PEG) and RGD, a peptide containing integrin-binding domain, compared to fibroblasts (164), suggesting that the use of nanotechnology could be helpful for treatments already used. Additionally, CAFs overexpress ER-α in tumor tissues of patients with CC, ER-α signaling promotes the expression of genes related to cell proliferation (*PDGF-C* and *EREG*), angiogenesis (*VEGF-A, VEGF-C*, *CTGF*, and *ANGPT1*), metastasis (*MMP-1* and *COL6A1*) and tumor growth (*FGF1*), making ER-α overexpressing CAFs an important therapeutic target (165).

**Figure 2:**
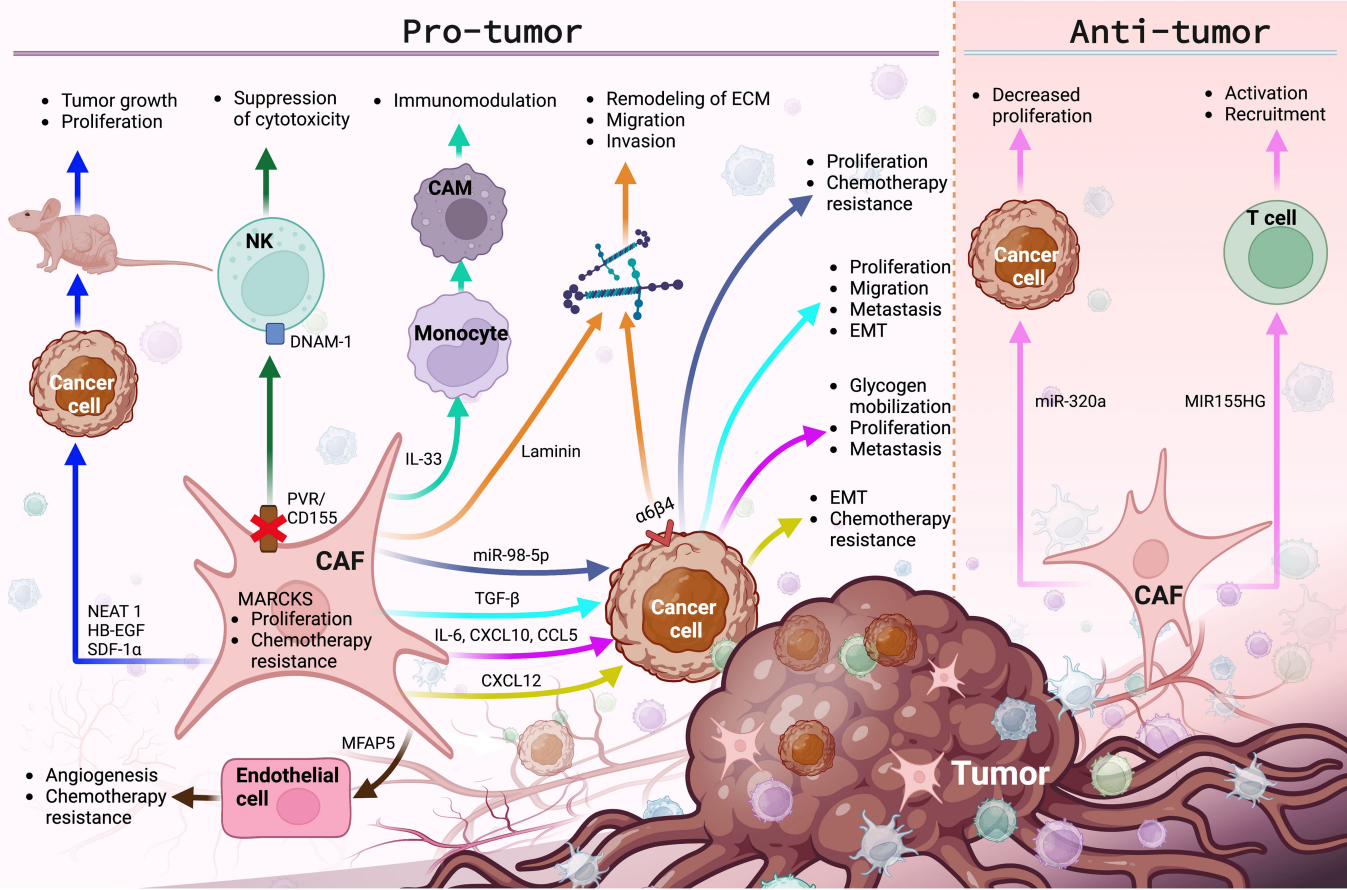
The main molecular functions and cellular events exerted by CAFs in the TME of gynecological cancers. CAFs promote various pro-tumor and anti-tumor events, by regulating the expression of membrane receptors and the secretion of growth factors, lncRNAs, miRNAs, interleukins, chemokines and ECM proteins, modulating cancer progression. Image created in BioRender.com and data sourced from (25, 28, 30, 84, 114, 117, 118, 121–123, 127, 133, 136, 137, 139, 152, 158–163, 165).

These findings confirm the participation of CAFs in the carcinogenesis and progression of gynecological cancers (**Table 1** and **Figure 2**), promoting that among the first ten cancers with the highest incidence and mortality worldwide are ovarian, endometrial, and cervical cancer (2).

**Table 1:** Events generated by CAFs and tumor effect in gynecological cancers.

Cancer	Events generated by CAFs	Tumor effect	References
*Ovarian*	Promotion of migration, invasion, EMT, and tumorigenesis through the activation of SMAD signaling pathway by exosomal TGF-β in OC cells	*PRO-TUMOR*	(30)
	Promotion of angiogenesis, tumor growth, invasion, and metastasis through the secretion of MFAP5	*PRO-TUMOR*	(84)
	ECM remodeling and promotion of metastasis by inducing the expression of MMP11 and MMP13 in OC cells through the secretion of TGF-β	*PRO-TUMOR*	(114)
	Induction of migration, proliferation, EMT, and metastasis by overexpression of NNMT	*PRO-TUMOR*	(116)
	Immunomodulation of TME promoting the polarization of monocytes to M2 type-like CAMs through the secretion of IL-33	*PRO-TUMOR*	(117)
	Generation of resistance to paclitaxel weakening blood vessels causing drug leakage before being released to OC cells	*PRO-TUMOR*	(118)
	Association with high recurrence in patients with OC	*PRO-TUMOR*	(120)
	Facilitation of metastasis through the expression of MARCKS	*PRO-TUMOR*	(121)
	Correlation with reduced overall survival and promotion of migration, invasion, and EMT	*PRO-TUMOR*	(122)
	Induction of EMT and cisplatin chemoresistance in OC patients by CXCL12	*PRO-TUMOR*	(123)
	Favoring the conversion of glycogen to glucose in OC cells to provide them with energy	*PRO-TUMOR*	(127)
	Increase of cell proliferation, inhibition of apoptosis, and promotion of cisplatin resistance in OC cells by miR-98-5p secretion	*PRO-TUMOR*	(133)
	Association with T cell activation, antigen processing, and presentation, and enriched infiltrate of immune cell anti-tumors within the tumor by the secretion of the lncRNA *MIR155HG*	*ANTI-TUMOR*	(136)
*Endometrial*	Promotion of EC progression by secreting exosomal lncRNA NEAT1	*PRO-TUMOR*	(25)
	Suppression of NK cell cytotoxicity by deregulating the expression of PVR/CD155	*PRO-TUMOR*	(137)
	Inhibition of cell proliferation by direct transfer of CAF-secreted exosomal miR-320a to EC cells	*ANTI-TUMOR*	(139)
	Promotion of migration, invasion, and EMT in EC cells by regulating the expression of PTTG	*PRO-TUMOR*	(146)
	Promotion of EC cells proliferation *via* PI3K/Akt and MAPK/Erk	*PRO-TUMOR*	(147)
	Promotion of proliferation, migration, invasion, and *in vivo* tumorigenesis by the secretion of the SDF-1α	*PRO-TUMOR*	(154)
*Cervical*	Increased migration and invasion of CC cells by secreting TGF-β	*PRO-TUMOR*	(28, 158, 159)
	Promotion of cell proliferation and tumor growth by secreting HB-EGF in CC patients, and association with carcinogenesis and disease progression	*PRO-TUMOR*	(160)
	Induction of tumor growth, angiogenesis, and lymph node metastasis in an animal model	*PRO-TUMOR*	(161)
	Remodeling of the ECM and promotion of cell invasion through laminin secretion	*PRO-TUMOR*	(162)
	Generation of lymph node metastasis and poor survival in CC patients by inducing the permeability of lymphatic endothelial barriers	*PRO-TUMOR*	(163)
	Promotion of the expression of genes related to cell proliferation, angiogenesis, metastasis, and tumor growth *via* ER-α	*PRO-TUMOR*	(165)

## Conclusion

CAFs play an important role in the TME, promoting tumorigenesis and malignant progression. Despite the considerable evidence about the pro-tumor role of CAFs in gynecological cancers, the design of personalized therapies directed at them must be careful due to the existence of evidence that proposes them as anti-tumor cells, although there are few reports that support this idea. In various cancers, clinical trials have been carried out where CAFs have been used as a therapeutic tool (166–168), nevertheless, few have fully reproduced the promising results obtained in the laboratory, which are necessary to include this cell type in cancer treatments. This could be a consequence of their heterogeneous origin, their different protein expression patterns and the pro-tumor and anti-tumor effects that they exert on the TME (169, 170). Despite this, CAFs are an ideal target to direct antineoplastic therapies; the meticulous point in this sense will be to direct blocking molecules specifically on molecules with pro-tumor effect and favor the expression of molecules with anti-tumor role, in gynecological cancer clinical trials with CAFs as a therapeutic target are practically non-existent. CAFs could stop being enemies in the battle against gynecological cancers, become allies and achieve longer progression-free survival and overall survival. Well-designed future clinical trials that provide conclusive evidence on the specific roles of CAF subpopulations within the TME and in the PMN are needed to translate the results found in basic research on CAFs into clinical practice and finally obtain clinical benefits.

## Author contributions

JV-P conceptualized the manuscript, JV-P, AR-d-A, LB-U, ML-T, and AP-S wrote it, and ML-T designed the figures. AR-d-A and AP-S critically reviewed it. AP-S led the activities. All authors contributed to the article and approved the submitted version.
